# Excess Protein O-GlcNAcylation Links Metabolic Derangements to Right Ventricular Dysfunction in Pulmonary Arterial Hypertension

**DOI:** 10.3390/ijms21197278

**Published:** 2020-10-01

**Authors:** Sasha Z. Prisco, Lauren Rose, Francois Potus, Lian Tian, Danchen Wu, Lynn Hartweck, Ruaa Al-Qazazi, Monica Neuber-Hess, Megan Eklund, Steven Hsu, Thenappan Thenappan, Stephen L. Archer, Kurt W. Prins

**Affiliations:** 1 Cardiovascular Division, Lillehei Heart Institute, University of Minnesota, Minneapolis, MN 55455, USA; szprisco@umn.edu (S.Z.P.); lerose@umn.edu (L.R.); hartw006@umn.edu (L.H.); eklun114@d.umn.edu (M.E.); tthenapp@umn.edu (T.T.); 2 Department of Medicine, Queen’s University, Kingston, ON K7L3N6, Canada; fp17@queensu.ca (F.P.); lian.tian@strath.ac.uk (L.T.); danchen.wu@queensu.ca (D.W.); 17rasa1@queensu.ca (R.A.-Q.); neuberm@queensu.ca (M.N.-H.); stephen.archer@queensu.ca (S.L.A.); 3 Division of Cardiology, Department of Medicine, Johns Hopkins University, Baltimore, MD 21287, USA; steven.hsu@jhmi.edu

**Keywords:** right ventricle, pulmonary hypertension, metabolism, post-translational modification, mitochondria

## Abstract

The hexosamine biosynthetic pathway (HBP) converts glucose to uridine-diphosphate-*N*-acetylglucosamine, which, when added to serines or threonines, modulates protein function through protein O-GlcNAcylation. Glutamine-fructose-6-phosphate amidotransferase (GFAT) regulates HBP flux, and AMP-kinase phosphorylation of GFAT blunts GFAT activity and O-GlcNAcylation. While numerous studies demonstrate increased right ventricle (RV) glucose uptake in pulmonary arterial hypertension (PAH), the relationship between O-GlcNAcylation and RV function in PAH is unexplored. Therefore, we examined how colchicine-mediated AMP-kinase activation altered HBP intermediates, O-GlcNAcylation, mitochondrial function, and RV function in pulmonary artery-banded (PAB) and monocrotaline (MCT) rats. AMPK activation induced GFAT phosphorylation and reduced HBP intermediates and O-GlcNAcylation in MCT but not PAB rats. Reduced O-GlcNAcylation partially restored the RV metabolic signature and improved RV function in MCT rats. Proteomics revealed elevated expression of O-GlcNAcylated mitochondrial proteins in MCT RVs, which fractionation studies corroborated. Seahorse micropolarimetry analysis of H9c2 cardiomyocytes demonstrated colchicine improved mitochondrial function and reduced O-GlcNAcylation. Presence of diabetes in PAH, a condition of excess O-GlcNAcylation, reduced RV contractility when compared to nondiabetics. Furthermore, there was an inverse relationship between RV contractility and HgbA1C. Finally, RV biopsy specimens from PAH patients displayed increased O-GlcNAcylation. Thus, excess O-GlcNAcylation may contribute to metabolic derangements and RV dysfunction in PAH.

## 1. Introduction

Right ventricular dysfunction (RVD) is the strongest predictor of mortality in pulmonary arterial hypertension (PAH) [1,2,3], however the molecular mechanisms that mediate RVD are incompletely defined. The most intensively characterized phenotypes of RVD are metabolic derangements [4], which includes marked increases in right ventricular glucose uptake in both preclinical models [5] and PAH patients [6,7,8]. While glucose is predominately metabolized via glycolysis to generate adenosine triphosphate, the hexosamine biosynthetic pathway (HBP) converts 2–3% of glucose to uridine diphosphate-*N*-acetylglucosamine (UDP-GlcNAc) [9]. UDP-GlcNAc can be used to post-translationally modify proteins, a process known as protein O-GlcNAcylation (O-GlcNAc) [9]. In addition to O-GlcNAcylation, UDP-GlcNAc is a sugar nucleotide donor for multiple other glycosylation events [9].

There are three key enzymes that can directly or indirectly regulate O-GlcNAcylation. First, the rate limiting enzyme for HBP flux is glutamine-fructose-6-phosphate amidotransferase (GFAT) [9]. GFAT is inhibited by AMP-kinase (AMPK) mediated phosphorylation, which blunts its activity and thus slows UDP-GlcNAc synthesis and thereby decreases O-GlcNAcylation [10,11]. The second key enzyme is O-linked β-N-*acetyl*glucosamine transferase (OGT). OGT catalyzes the addition of GlcNAc to serine or threonine residues in proteins and thus OGT is responsible for O-GlcNAcylation [9]. The final enzyme that regulates O-GlcNAcylation is O-GlcNAcase (OGA). OGA removes GlcNAc from proteins and thus reverses O-GlcNAcylation [9]. While O-GlcNAcylation is elevated in diabetic cardiomyopathy [12,13,14] and chronic excess O-GlcNAcylation causes mitochondrial dysfunction [14,15,16], the role of O-GlcNAcylation in RVD in PAH is unexplored. Importantly, previous work shows that one of the major causes of RVD in PAH relates to mitochondrial metabolic dysfunction [17,18,19]. Moreover, we and others have demonstrated that metabolic derangements are present in both experimental and human PAH RVs [20,21,22,23]. Thus, excess O-GlcNAcylation may promote the dysfunctional mitochondrial phenotype that is repeatedly observed in the PAH right ventricle.

Here, we investigated the hypothesis that excess O-GlcNAcylation promotes mitochondrial dysfunction and RVD in PAH. We tested this hypothesis by examining the relationship between HBP intermediate levels, O-GlcNAcylation, and RV function in the monocrotaline (MCT) and pulmonary artery-banded (PAB) rat models of RV pressure overload that exhibit different degrees of RVD severity, severe (MCT) versus mild (PAB). We then examined the therapeutic implications of AMP-kinase activation, using colchicine, on HBP intermediate levels, O-GlcNAcylation, the right ventricle’s metabolic signature and function in both models. Finally, in PAH patients we examined the relationship between diabetes, a condition characterized by excess cardiac O-GlcNAcylation [12,13,14], and right ventricular function. We discovered that O-GlcNAcylation is increased in both experimental and human PAH and predicts right ventricular dysfunction. Furthermore, colchicine-mediated AMP-kinase activation results in a reduction in O-GlcNAcylation, augments mitochondrial function, and improves right ventricular function in experimental PAH only in the MCT model, which uniquely had significant dysregulation of the HBP and total protein O-GlcNAcylation. Importantly, colchicine did not significantly alter OGT and OGA levels, suggesting its effect on AMP-kinase was the most important driver of O-GlcNAcylation regulation.

## 2. Results

We first characterized the right ventricular phenotype of PAB and MCT rats. MCT rats had more pronounced right ventricular dysfunction as demonstrated by a significantly lower TAPSE (Supplemental Figure S1A). Both PAB and MCT rats had right ventricular hypertrophy (RVH), but the RVH was more pronounced in MCT rats (Supplemental Figure S1B). Collectively, these results demonstrate MCT rats have more severe right ventricular dysfunction than PAB rats, which allows for analysis of differential contributions of the O-GlcNAcylation pathway to preclinical right ventricular dysfunction.

**Figure 1:**
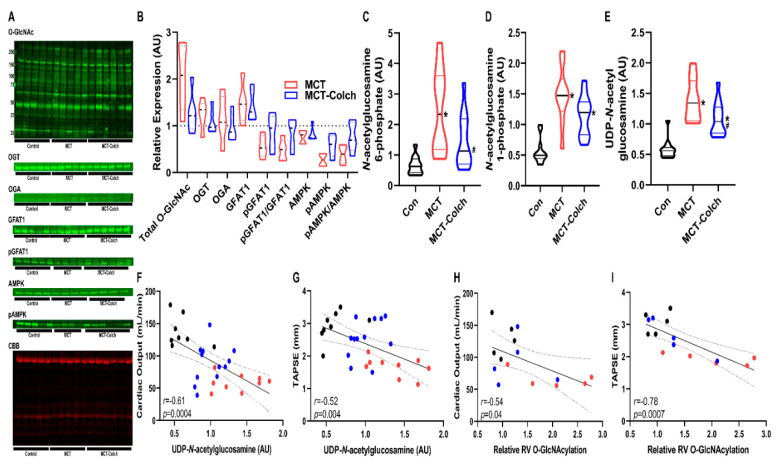
Colchicine activates AMPK, which induces GFAT1 phosphorylation, reduces hexosamine biosynthetic pathway (HBP) intermediate levels, and improves right ventricular function in MCT rats. (**A**) Representative Western blots of right ventricular extracts and quantification (**B**) from control, MCT, and MCT-Colch rats (*n* = 4–6 animals per group). O-GlcNAc (MCT: 2.0 ± 0.7 vs. MCT-Colch: 1.3 ± 0.4 fold increase compared to control), OGT (MCT: 1.2 ± 0.3 vs. MCT-Colch: 1.1 ± 0.3 fold increase compared to control), OGA (MCT: 1.2 ± 0.5 vs. MCT-Colch: 1.0 ± 0.3 fold increase compared to control), GFAT1 (MCT: 1.5 ± 0.4 vs. MCT-Colch: 1.4 ± 0.3 fold increase compared to control), phosphoGFAT1 (MCT: 0.6 ± 0.2 vs. MCT-Colch: 0.9 ± 0.4 relative expression compared to control), pGFAT1/GFAT1 (MCT: 0.5 ± 0.2 vs. MCT-Colch: 0.8 ± 0.3 relative expression compared to control), AMPK (MCT: 0.7 ± 0.1 vs. MCT-Colch: 0.8 ± 0.1 relative expression compared to control), pAMPK (MCT: 0.3 ± 0.1 vs. MCT-Colch: 0.6 ± 0.2 relative expression compared to control), pAMPK/AMPK (MCT: 0.4 ± 0.2 vs. MCT-Colch: 0.8 ± 0.3 relative expression compared to control). CBB gel was used to normalize protein load and highlights control samples had higher load than MCT and MCT-Colch. Metabolomic quantification of right ventricular levels of *N*-acetylglucosamine-6-phosphate (**C**), *N*-acetylglucosamine-1-phosphate (**D**), and UDP-GlcNAc (**E**) in control, MCT, and MCT-Colch right ventricular samples (*n* = 12 animals per group). There are significant inverse relationships between right ventricular UDP-GlcNAc levels and cardiac output (*r* = −0.61, *p* = 0.0004) (**F**) and TAPSE (*r* = −0.52, *p* = 0.004) (**G**). Total O-GlcNAcylation displayed significant negative relationships with cardiac output (*r* = −0.54, *p* = 0.04) (**H**) and TAPSE (*r* = −0.78, *p* = 0.0007) (**I**). * indicates significantly different from control and # indicates significantly different from MCT as determined by one-way ANOVA with Tukey post-hoc analysis. Black dots: control, blue dots: MCT-Colch, and red dots: MCT. CBB: Coomassie brilliant blue. Tissue was harvested 4 weeks after MCT injection.

Next, we determined how inhibition of protein O-GlcNAcylation, achieved by colchicine-induced AMPK activation and subsequent GFAT1 phosphorylation, altered RV function. In MCT rats, colchicine treatment increased the ratio of phosphorylated to total AMPK (MCT-Colch: 0.8 ± 0.3 vs. MCT: 0.4 ± 0.2 relative expression compared to control). This was associated with a higher phospho-GFAT1/GFAT1 ratio (MCT-Colch: 0.8 ± 0.3 vs. MCT: 0.5 ± 0.2 relative expression compared to control) (Figure 1A,B). This resulted in a reduction in total O-GlcNAcylation (MCT: 2.0 ± 0.7 vs. MCT-Colch: 1.3 ± 0.4 fold increase compared to control). Importantly, colchicine did not alter the slight upregulation of OGT that was observed in MCT right ventricles (MCT: 1.2 ± 0.3 vs. MCT-Colch: 1.1 ± 0.3 fold increase compared to control). Colchicine treatment induced reductions in right ventricular HBP intermediates (Figure 1C–E), which was associated with improved right ventricular function, evident in the significant negative relationships between right ventricular UDP-GlcNAc levels and cardiac output (*r* = −0.61, *p* = 0.0004) and TAPSE (*r* = −0.52, *p* = 0.004) (Figure 1F,G). Furthermore, total right ventricular O-GlcNAcylation also displayed significant negative relationships with cardiac output (*r* = −0.54, *p* = 0.04) and TAPSE (*r* = −0.78, *p* = 0.0007) (Figure 1H,I). These results demonstrate that increased O-GlcNAcylation and HBP intermediate levels in MCT rats were associated with worse right ventricular function, and suggest that the reduction in O-GlcNAcylation and HBP intermediates caused by colchicine treatment helped improve right ventricular function

**Figure 2:**
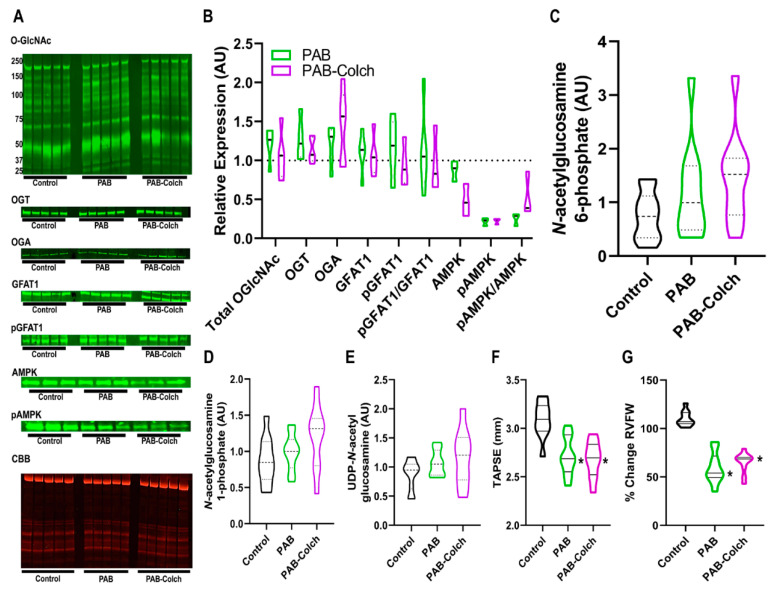
Colchicine has minimal effects on HBP intermediate levels, O-GlcNAcylation, and right ventricular function in PAB rats. (**A**) Representative Western blots and subsequent quantification (**B**) of protein abundance in right ventricular extracts from control, PAB, and PAB-Colch rats (*n* = 3–5 animals per group). O-GlcNAc (PAB: 1.2 ± 0.2, PAB-Colch 1.1 ± 0.3 fold increase versus control), OGT (PAB: 1.3 ± 0.3, PAB-Colch 1.1 ± 0.2 fold increase versus control), OGA (PAB: 1.2 ± 0.3, PAB-Colch 1.4 ± 0.4 fold increase versus control), GFAT1 (PAB: 1.1 ± 0.3, PAB-Colch 1.1 ± 0.3 fold increase versus control), phosphoGFAT1 (PAB: 1.2 ± 0.4, PAB-Colch 1.0 ± 0.3 relative expression compared to control), pGFAT1/GFAT1 (PAB: 1.1 ± 0.6, PAB-Colch 1.0 ± 0.4 relative expression compared to control), AMPK (PAB: 0.9 ± 0.1, PAB-Colch 0.5 ± 0.2 relative expression compared to control), pAMPK (PAB: 0.2 ± 0.1, PAB-Colch 0.2 ± 0.05 relative expression compared to control), pAMPK/AMPK (PAB: 0.3 ± 0.1, PAB-Colch 0.5 ± 0.3 relative expression compared to control). Quantification of levels of *N*-acetylglucosamine-6-phosphate (**C**), *N*-acetylglucosamine-1-phosphate (**D**), and UDP-GlcNAc (**E**) in the right ventricle of control, PAB, and PAB-Colch rats (*n =* 10 for each group). There were no significant changes in O-GlcNAcylation and HBP intermediates when the three groups were compared. Quantification of right ventricular function shows colchicine did not improve TAPSE (PAB: 2.7 ± 0.2 mm, PAB-Colch: 2.7 ± 0.2 mm) (**F**) or RV free wall thickening (PAB: 59 ± 16%, PAB-Colch: 65 ± 11%) (**G**) in PAB rats. (*) indicates significantly different from control as determined by one-way ANOVA with Tukey post-hoc analysis. Tissue was harvested 8 weeks after sham or PAB procedure. CBB: Coomassie brilliant blue.

In PAB rats the ratio of phosphorylated GFAT1/GFAT1 was not significantly dysregulated in PAB or PAB-Colch right ventricles (Figure 2A,B). However, colchicine increased the ratio of phosphorylated AMPK/AMPK, but there was a reduction in total AMPK in the colchicine treated group (Figure 2A,B). Consistent with the lack of excess activation of the HBP, colchicine treatment did not alter the levels of HBP intermediates in PAB right ventricles (Figure 2C–E), suggesting GFAT1 activity was not significantly altered in PAB or PAB-Colch right ventricles. Finally, colchicine did not improve right ventricular function in PAB rats (Figure 2F,G). These results show that although colchicine treatment activated AMPK in PAB RVs, it did not augment right ventricular function in a model in which HBP intermediates and O-GlcNAcylation are not dysregulated.

We also examined O-GlcNAcylation in the left ventricle of MCT and PAB rats to determine if there were chamber specific differences in the pathway. Overall, there were no significant differences in O-GlcNAcylation in the left ventricle in MCT versus control rats. MCT rats had lower levels of OGT (0.8 ± 0.1 relative abundance) and OGA (0.6 ± 0.2 relative abundance) but no changes in GFAT1 (Supplemental Figure S2). In PAB left ventricular extracts, O-GlcNAcylation was not altered, but again both OGT (0.8 ± 0.1 relative abundance) and OGA (0.6 ± 0.1 relative abundance) levels were reduced while GFAT was not changed (Supplemental Figure S2). These results suggest changes in O-GlcNAcylation are restricted to the failing right ventricle.

**Figure 3:**
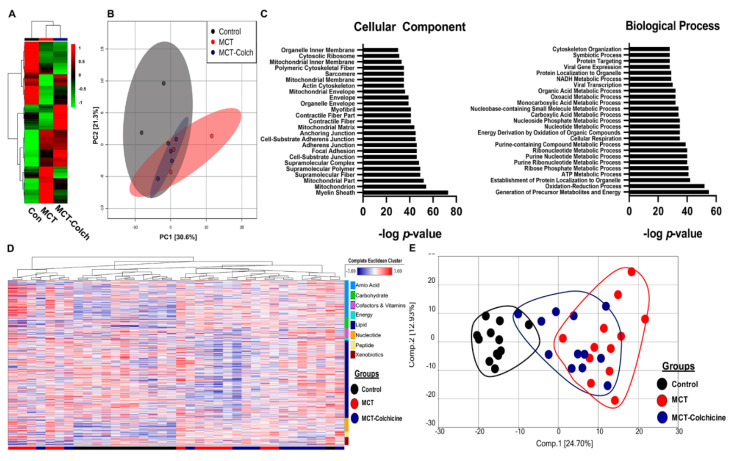
Proteomics and metabolomics link excess O-GlcNAcylation to metabolic dysregulation in the right ventricle. (**A**) Unbiased hierarchical cluster analysis of O-GlcNAcylated immunoprecipitation proteomics experiment. (**B**) PCA plot revealed differences in O-GlcNAcylation signatures in control, MCT, and MCT-Colch right ventricles. (**C**) Gene ontology assessment of cellular component and biological process of O-GlcNAcylated proteins from mass spectrometry experiment. (**D**) Hierarchical cluster analysis of metabolites from right ventricular extracts. Bars at bottom of graph indicate group. Black: control, Red: MCT, and Blue: MCT-Colchicine. On right side of graph, metabolites are clustered based on their classification. (**E**) PCA plot of total metabolomic data reveals MCT-Colch rats display a metabolic signature that is an intermediate of control and MCT. Tissue was harvested 4 weeks after MCT injection.

**Figure 4:**
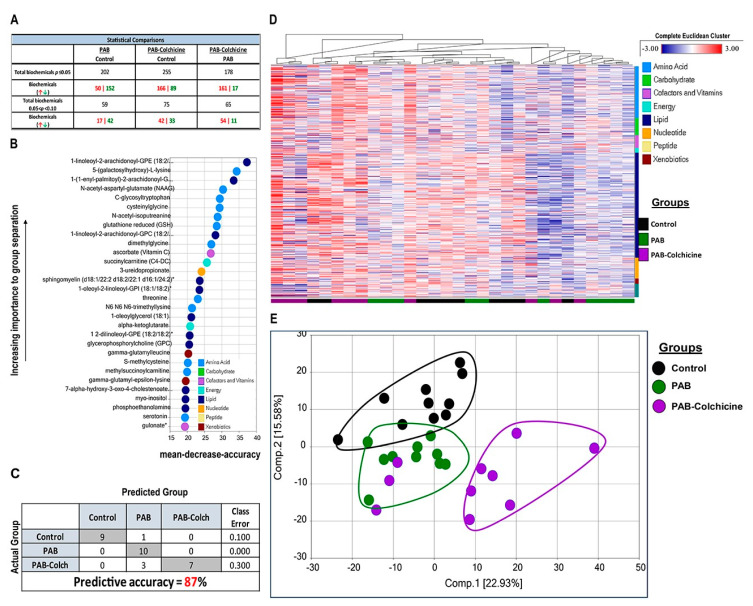
Effect of colchicine treatment on metabolomic signature in PAB rats. (**A**) Table depicting the overall differences in metabolites in control, PAB, and PAB-Colch right ventricles. (**B**) Biochemical importance plot revealing the top 30 ranking biochemicals that differentiates experimental groups. (**C**) Random Forest Confusion Matrix shows analysis of metabolomics data results in an 87% predictive accuracy. (**D**) Hierarchical clustering analysis of metabolites. (**E**) Principal component analysis of total metabolomic data reveals colchicine did not normalize the metabolic signature in PAB rats. Tissue was harvested 8 weeks after sham or PAB procedure.

To understand how manipulation of O-GlcNAcylation regulated right ventricular function, we performed quantitative proteomics analysis of immunoprecipitated O-GlcNAcylated proteins from control, MCT, and MCT-Colch right ventricular extracts. We identified 522 total proteins from right ventricular extracts, with quantitative information available on 509 proteins. Unbiased hierarchical analysis revealed differences in global O-GlcNAcylation between the three experimental groups (Figure 3A). Principal component analysis (PCA) demonstrated differences in the O-GlcNAcylation proteome signature between control and MCT while MCT-Colch displayed an intermediate signature (Figure 3B). Gene ontology analysis of O-GlcNAcylated proteins revealed their imputed impact on multiple metabolic processes with an enrichment of proteins predicted to be located in the mitochondria (Figure 3C). When we examined the 10 most differentially regulated mitochondrial proteins (HADHA, SLC25A3, CHCHD3, ATP5F1A, IMMT, HADHB, NDUFS1, UQCRC2, MRPL34, SUCLG2), we observed a consistent trend that colchicine reduced the levels of the proteins in the immunoprecipitation, suggesting a reduction in protein O-GlcNAcylation (Table 1). Importantly, eight of the 10 proteins we identified are known to be O-GlcNAcylated (Table 1). Next, we performed global metabolomics analysis to quantify the effect of O-GlcNAcylation on metabolic and mitochondrial function. Overall, there were 392 metabolites that were differentially regulated in MCT with 218 elevated and 174 reduced when compared to control (Supplemental Figure S3). MCT-Colch rats had 169 metabolites that were significantly different when compared to MCT (76 elevated and 93 reduced) (Supplemental Figure S3). Hierarchical cluster analysis demonstrated global differences in the metabolic profiles of the three experimental groups (Figure 3D). Finally, PCA revealed control and MCT rats displayed a distinct right ventricular metabolic signature while MCT-Colch rats exhibited a metabolic signature that was an intermediate between control and MCT (Figure 3E). In particular, one MCT-Colch animal had a control signature, three had signatures that were distinct from control and MCT, and eight had signatures that overlapped with MCT. In PAB right ventricles, colchicine treatment did not normalize the right ventricular metabolic signature (Figure 4) as three PAB-Colch had a metabolic signature that overlapped with PAB and seven had signatures that were distinct from both sham and PAB.

To confirm our proteomics analysis demonstrating MCT right ventricular mitochondrial proteins exhibited excess O-GlcNAcylation, we conducted cellular fractionation experiments. MCT rats had more O-GlcNAcylation in mitochondrial extracts (2.0 ± 0.5 fold increase relative to control rats), and this was attenuated by colchicine treatment (1.3 ± 0.4 fold increase relative to control rats) (Supplemental Figure S4). In the cytoplasmic extracts, there were no differences in O-GlcNAcylation in MCT and MCT-Colch rats (Supplemental Figure S4).

To examine the direct effect of colchicine on mitochondrial function and O-GlcNAcylation, we performed Seahorse microplanimetry analysis on H9c2 cardiomyocytes grown in high glucose to promote excess O-GlcNAcylation and recapitulate the failing RV in vitro. Colchicine augmented oxygen consumption rate (OCR) (Supplemental Figure S5A) while no significant changes in the extracellular acidification ratio, a measure of glycolysis, were observed (Supplemental Figure S5B). In particular, colchicine treatment increased maximal OCR (Supplemental Figure S5C) and spare OCR (Supplemental Figure S5D), while decreasing proton leak (Supplemental Figure S5E). Importantly, colchicine induced AMPK phosphorylation (1.8 ± 0.3 fold increase) and reduced O-GlcNAcylation (0.8 ± 0.3 relative expression) (Supplemental Figure S5F,G). Collectively, these results demonstrate colchicine treatment directly reduces O-GlcNAcylation and augments mitochondrial function in a cardiomyocyte cell line.

**Figure 5:**
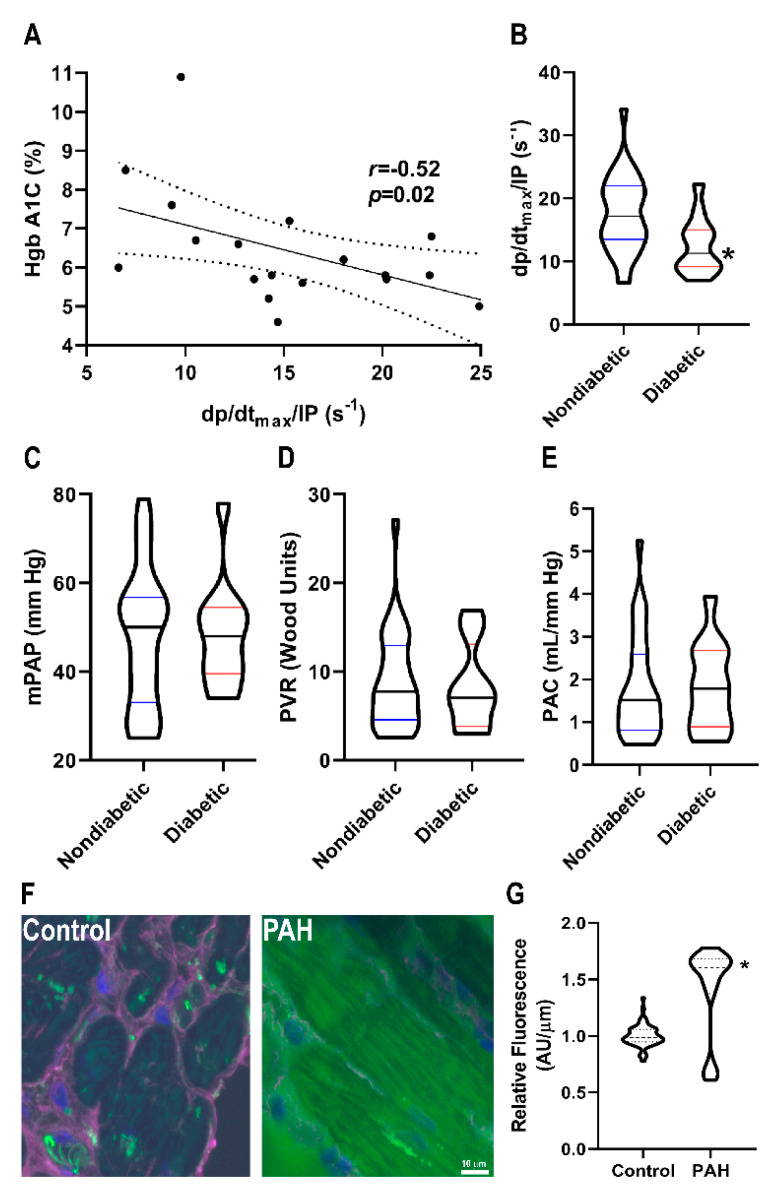
Relationship between O-GlcNAcylation and RVD in PAH patients. (**A**) Correlation between HgbA1C and right ventricular contractility in PAH patients. There is a significant negative relationship between HgbA1C and right ventricular contractility (*r* = −0.52, *p* = 0.02) (**B**) Diabetic patients have lower right ventricular contractility than nondiabetic PAH patients (12.5 ± 4.5 s^−1^ vs. 17.5 ± 6.0 s^−1^, *p* = 0.01). There were no significant differences in pulmonary vascular disease severity in diabetic versus nondiabetic patients as quantified by mean pulmonary artery pressure (48 ± 12 vs. 48 ± 16 mmHg, *p* = 0.37) (**C**), pulmonary vascular resistance (8.3 ± 4.9 vs. 8.9 ± 5.5 Wood units, *p* = 0.68) (**D**), and pulmonary arterial compliance (1.8 ± 1.0 vs. 1.8 ± 1.2 mL/mm Hg, *p* = 0.64) (**E**). (**F**) Representative confocal micrographs of right ventricular sections stained with succinylated wheat germ agglutinin (WGA). (**G**) Quantification of succinylated WGA signal intensity in right ventricular cardiomyocytes from two control (*n* = 63 total cells analyzed) and two PAH biopsy specimens (*n* = 55 total cells analyzed). Green: Succinylated WGA, Blue: DAPI, and Magenta: WGA. * indicates significantly different as determined by *t*-test in (**B**) or Mann–Whitney U-test in (**G**).

To further explore the potential link between O-GlcNAcylation and right ventricular function, we examined the effects of diabetes on right ventricular function in PAH patients (Table 2) because diabetes is a disease of excess cardiomyocyte O-GlcNAcylation [12,13,14]. There was a significant, negative relationship between right ventricular contractility and HgbA1C levels in PAH patients (*r* = −0.52, *p* = 0.02) (Figure 5A). Moreover, diabetic patients had reduced right ventricular contractility (12.5 ± 4.5 s^−1^ vs. 17.5 ± 6.0 s^−1^, *p* = 0.01) as compared to nondiabetic patients (Figure 5B). This relationship was independent of pulmonary vascular disease severity as there were no significant differences in mean pulmonary arterial pressure (48 ± 12 mmHg vs. 48 ± 16 mmHg, *p* = 0.95), pulmonary vascular resistance (8.3 ± 4.9 Wood units vs. 9.0 ± 5.5 Wood units, *p* = 0.72), and pulmonary arterial compliance (1.8 ± 1.0 mL/mmHg vs. 1.8 ± 1.2 mL/mmHg, *p* = 0.98) (Figure 5C–E) between diabetics and nondiabetics. Finally, to directly examine O-GlcNAcylation in human PAH right ventricular sections, we performed quantitative immunofluorescence analysis, and observed a significant increase in cardiomyocyte O-GlcNAcylation signal in PAH samples as compared to control subjects (Figure 5F–I).

## 3. Discussion

Right ventricular dysfunction is the leading cause of death in PAH patients [25], and thus finding therapies to improve right ventricular function is paramount for combating this deadly disease. Mitochondrial metabolic pathways, notably impaired glucose oxidation and increased uncoupled glycolysis, are well established contributors of right ventricular dysfunction in PAH [18,25]. Under these conditions, ATP production is supported by heightened right ventricular glucose uptake, which can be visualized by 18^F^-fluorodeoxyglucose positron emission tomography [5,6,7,8]. While the Warburg mechanism is important for compensating for mitochondrial dysfunction to generate ATP [18,25], studies have not examined the potential additional adverse effects of increased intracellular glucose, which would accompany this metabolic shift. Here, we show increased glucose flux through the HBP has an adverse effect on the right ventricle via excess O-GlcNAcylation of mitochondrial proteins, which independently reinforces metabolic abnormalities in the PAH right ventricle. In particular, we show that increased expression of GFAT1 results in elevated levels of HBP intermediates, excess O-GlcNAcylation, and more severe RVD in a well validated preclinical model of RV pressure overload and PAH, the MCT rat model. Importantly, this process is specific to the failing right ventricle, as the left ventricle is spared in MCT rats. Moreover, this molecular signature is not observed in a pure right ventricular pressure overload model with preserved right ventricular function that is induced by PAB. Finally, the beneficial effects of colchicine, which are in-part mediated by activation of AMPK and inhibition of excess O-GlcNAcylation, illustrate the potential for targeting this pathway pharmacologically.

We show that activation of AMPK with colchicine reduces O-GlcNAcylation which results in augmented right ventricular function (Figure 1). To help understand this phenomenon, we used proteomics to define and quantify proteins that undergo differential O-GlcNAcylation. Gene ontology analysis of our proteomic data predicts an enrichment of mitochondrial proteins with multiple metabolic functions that are O-GlcNAcylated in MCT right ventricles (Figure 3), which is corroborated by cellular fractionation studies (Supplemental Figure S4). Previous studies demonstrate chronic excess O-GlcNAcylation is associated with impaired mitochondrial function [14,15,16], and thus increased mitochondrial O-GlcNAcylation could cause mitochondrial dysfunction in the MCT right ventricle. This hypothesis is consistent with our observations that colchicine reduces O-GlcNAcylation and augments oxygen consumption in H9c2 cells and that colchicine reduces HBP intermediate levels and O-GlcNAcylation and partially normalizes the right ventricular metabolic signature in MCT rats. However, there is cross-talk between AMPK and O-GlcNAcylation so the relationship between AMPK, O-GlcNAcylation, and right ventricular function is likely more complex. For instance, AMPK activation can promote nuclear localization of OGT and thus modulate which proteins are O-GlcNAcylated [26]. Furthermore, O-GlcNAcylation of the gamma subunit of AMPK increases AMPK activity [26]. Clearly, the intersections between AMPK, O-GlcNAcylation, and metabolic function will require further investigation in the future.

While the role of O-GlcNAcylation in RVD in PAH has not been explicitly explored previously, there are suggestions in the literature that excess O-GlcNAcylation contributes to RVD. We previously demonstrated that use of 6-diazo-5-oxo-L-norleucine (DON), an inhibitor of GFAT [27] and glutaminolysis [17], acutely increases cardiac output in MCT hearts but not PAB hearts in the working heart model [17]. Moreover, chronic treatment of MCT rats with DON improves right ventricular function and augments treadmill exercise capacity [17]. These results are congruent with our current findings as excess O-GlcNAcylation is found in MCT and not PAB right ventricle, and thus inhibiting GFAT1 pharmacologically may only be useful in states of excess O-GlcNAcylation. Certainly, the ability of DON to regulate glutaminolysis also contributes to its beneficial effects on the failing right ventricle. Furthermore, there is an association between O-GlcNAcylation and worse outcomes in PAH patients. O-GlcNAcylation levels in red blood cells of PAH patients are higher than control patients [28], and elevated OGT levels in red blood cells is associated with clinical worsening as defined by hospitalization, lung transplant, or death [28]. Thus, our data are consistent with previous publications and in summation these data suggest a pathological role of excess O-GlcNAcylation in RVD in PAH.

Heightened right ventricular glucose uptake in PAH patients is documented by several groups, and there is a clear inverse relationship between right ventricular glucose uptake and right ventricular systolic function [6,7,8]. While increased glucose uptake may be a biomarker of metabolic derangements in the right ventricle, our results suggest that elevated glucose uptake could directly contribute to metabolic dysfunction in the right ventricle via excess O-GlcNAcylation. Thus, it is conceivable that O-GlcNAcylation can further promote mitochondrial dysfunction resulting in impaired right ventricular contractility, which could help explain the consistent clinical observation that elevated right ventricular glucose uptake is associated with RV dysfunction in PAH patients.

There are reports that diabetes results in worse right ventricular function in both PAH and other pathological states. First, a study of 113 idiopathic or hereditary PAH patients from Vanderbilt University showed that diabetic patients have lower right ventricular stroke work index despite having a trend for less severe pulmonary vascular disease [29]. The diabetic PAH patients in this study also had increased mortality [29]. In a study that evaluated right ventricular dysfunction post ST elevation myocardial infarction (STEMI), the presence of diabetes was a multivariate predictor of right ventricular dysfunction post STEMI [30]. Moreover, patients with a HgbA1C ≥ 7.0 were more likely to develop right ventricular dysfunction post STEMI than those with HgbA1C < 7.0 [30]. The results of these two studies are consistent with our findings that diabetic PAH patients have lower right ventricular contractility (Figure 5), and our results suggest that excess O-GlcNAcylation could contribute to right ventricular dysfunction in multiple disease states.

Previous work demonstrated significant reductions in acyl-carnitine levels in the right ventricle in PAH patients [19]. In our metabolomics analysis, we also observed lower levels of nearly all acyl-carnitines in both MCT and PAB right ventricles (Supplemental Tables S1 and S2). Interestingly, MCT-Colch right ventricles had similar reductions in acyl-carnitines as MCT right ventricles, but MCT-Colch rats have better right ventricular function. Perhaps, dysregulation of acyl-carnitines is an early metabolic change in right ventricular pressure overload, and normalization of acyl-carnitine metabolism is not completely necessary to augment right ventricular function.

While we previously documented colchicine combats pathological microtubule remodeling and augments right ventricular function in MCT rats [31], we did not see significant improvements in right ventricular function in PAB rats treated with colchicine (Figure 2). There are potential explanations for this finding. First, the relative increase in tubulin, the individual subunits of microtubules, in PAB (Supplemental Figure S6) are less than what we observed in MCT rats [31], and there may be a threshold of microtubule remodeling needed for a pathological response. Second, it is possible that chronic colchicine treatment and microtubule depolymerization could have adverse effects on cardiac function as a previous manuscript showed high dose colchicine causes cardiac conduction abnormalities, however at much higher doses than we used [32]. While there are strong data linking microtubule remodeling to impaired cardiac contractility [33,34,35], perhaps the less prominent microtubule phenotype of PAB rats cannot be enhanced with colchicine because microtubule remodeling is not the driving force of this mild right ventricular dysfunction phenotype. Furthermore, the MCT right ventricle has more of an inflammatory phenotype than the PAB right ventricle [36], which may also help explain the lack of efficacy of colchicine in the PAB rats.

Consistent with our study, there are data that other AMP-kinase activating drugs have beneficial effects in the right ventricle in PAH. Metformin mitigates pulmonary vascular remodeling and augments cardiac output in multiple models of preclinical PAH [37]. Moreover, a study in left ventricular pressure overloaded mice shows metformin blunts protein O-GlcNAcylation via AMP-kinase activation resulting in a reduction in pathological left ventricular hypertrophy [10]. Interestingly, a clinical trial examining the utility of metformin in PAH patients is ongoing (NCT03617458), and right ventricular function, as determined by cardiac magnetic resonance imaging, will be one of the end-points analyzed in this trial. Moreover, the peroxisome proliferator-activation receptor gamma (PPARγ) pioglitazone can activate AMP-kinase, and a recent study demonstrated pioglitazone directly enhances right ventricular function in Sugen-hypoxia PAH rats [21]. Thus, there are multiple lines of evidence that activation of AMP-kinase augments right ventricular function, and modulation of O-GlcNAcylation may contribute to these findings.

## 4. Materials and Methods

### 4.1. Rat Models of RV Pressure Overload

Monocrotaline (MCT) PAH was induced by subcutaneous injection of MCT (Sigma-Aldrich, St. Louis, MO, USA) at a dose of 60 mg/kg in adult male Sprague-Dawley rats. For pulmonary artery banding, adult male Sprague-Dawley rats were anesthetized with 3% isoflurane and intubated. The main pulmonary artery (PA) was dissected from the ascending aorta via a left-sided thoracotomy. A 1.3-mm diameter needle was placed parallel to the main PA and ligated with a 4-0 silk suture. The needle was withdrawn to create a fixed stenosis as previously described [17]. Control rats for MCT received an injection of phosphate buffered saline (PBS) and control rats for PAB underwent a sham operation. Experiments were conducted in accordance with published guidelines of the Canadian Council of Animal Care and approved by Queen’s University Animal Care Committee. The animal protocol used in this work was evaluated and approved by Queen’s University Animal Care Committee (Protocol 2017-1714).

### 4.2. Colchicine Treatment

Filter-sterilized colchicine (Sigma-Aldrich) dissolved in PBS at a dose of 0.5 mg/kg three times per week was given to MCT rats 1 week after MCT injection for 3 weeks and then animals were analyzed. For PAB rats, colchicine treatment started 4 weeks after PAB, animals were treated for 4 weeks, and then were analyzed.

### 4.3. Western Blot Analysis

Immunoblots were performed on RV and LV specimens as described [31] using the Odyssey Infrared Imaging system (Li-Cor, Lincoln, NE, USA). Post transfer SDS-PAGE gels were stained with Coomassie brilliant blue and imaged at the 700-nm wavelength on the Odyssey Imaging system as the loading control, with the band corresponding to the myosin heavy chain used as the reference [31,38].

### 4.4. Antibodies

Antibodies used in study are listed in Supplemental Table S3. For the phospho-GFAT Western blots, we quantified the band at the dimer molecular weight as this protein functions as a dimer biologically [39] and the monomer size was not detected.

### 4.5. Global Metabolomics

Frozen RV free wall specimens were processed by Metabolon Inc. (Durham, NC, USA) for global metabolomics profiling. For the MCT experiments, control, MCT, and MCT treated with colchicine specimens were analyzed (*n* = 12 animals per group). For the PAB analysis, sham operated, PAB, and PAB treated with colchicine specimens were analyzed (*n* = 10 animals per group)

### 4.6. Rodent Echocardiography

Echocardiography was performed using a Vevo2100 ultrasound system with a 37.5-MHz transducer (Visual Sonics Inc, Toronto, ON, Canada) as previously described [31]. M-mode and 2-D modalities were applied to measure RV free wall (RVFW) thickness during end diastole and end systole and tricuspid annular plane systolic excursion (TAPSE). PA diameter was measured at the level of the pulmonary outflow tract during midsystole. Pulsed-wave Doppler was used to measure PA flow velocity time integral [40]. Cardiac output was calculated as previously described [40].

### 4.7. O-GlcNAcylated Protein Immunoprecipitation (IP)

Frozen RV samples were pulverized and incubated in solubilization buffer (0.025M Tris, 0.15M sodium chloride, 0.001M ethylenediaminetetraacetic acid, 1% NP40, 5% glycerol, pH 7.4) supplemented with mammalian protease inhibitor (Sigma-Aldrich) and the OGA inhibitor O-(2-acetamido-2-deoxy-D-glucopyranosylidenamino) N-phenylcarbamate (PUGNAc, Sigma-Aldrich) (100 µM). Protein concentration was determined using a BCA kit and then 2.5 mg of total protein extract was incubated with the pan O-GlcNAcylation monoclonal antibody RL2 (ThermoScientific) covalently linked to metallic beads (Pierce Co-Immunoprecipitation kit) overnight at 4 °C. The beads were washed with solubilization buffer twice and once with H_2_O. Protein was eluted from antibodies using acidic solution. Neutralization buffer was added to solutions prior to proceeding with mass spectrometry analysis. Raw proteomics data is supplied in Supplemental Table S4.

### 4.8. Quantitative Mass Spectrometry

A 32 µL aliquot of each IP sample was run into a BioRad 10% Criterion^TM^ Tris-HCl gel. Then the gel was fixed with 40% ethanol and 10% acetic acid. The gel was washed with LC-MS grade water and then stained with Thermo Scientific’s Imperial Protein stain and de-stained with LC-MS grade water overnight. Equal gel area regions for each sample were excised and subjected to in-gel proteolytic digestion as described previously [41] with the following difference: during the alkylation step, 55 mM iodoacetamide was used instead of 55 mM methyl methanethiosulfonate. Post digestion, each sample was then cleaned with an MCX STAGE tip [42]. Eluates were vacuum dried and resuspended in 20 µL 0.1 M triethylammonium bicarbonate, pH 8.5. A 19 µL aliquot of each sample was labeled with TMT10plex™ isobaric label reagent (Thermo Scientific, Waltham, MA, USA). After labeling, all 10 samples were mixed together in an equal ratio and the multiplexed sample was cleaned with a 3 cc Sep-Pak C18 solid phase extraction cartridge (Waters Corporation, Milford, MA, USA) and the eluate was dried in vacuo. The cleaned sample was run on the Thermo Fusion. LC-MS data was acquired for each sample using an Easy-nLC 1000 HPLC (Thermo Scientific) in tandem with an Orbitrap Fusion (Thermo Scientific). Peptides were loaded directly onto a 75 cm × 100-µm internal diameter fused silica PicoTip Emitter (New Objective, Woburn, MA, USA) packed in-house with ReproSil-Pur C18-AQ. The column was mounted in a nanospray source directly in-line with an Orbitrap Fusion mass spectrometer (Thermo Scientific). Gas phase fractionation was employed via multiple LC-MS acquisition runs on the same sample to survey mass spectra on windows of 380–680, 680–980, 980–1280, 1280–1580, 380–1580 *m*/*z* with a resolution of 60,000 at 100 *m*/*z* with automatic gain control, 250-ms min injection time and lock mass at 445.1200 *m*/*z* (polysiloxane). The 12 most intense ions (2–7 charged state) from the full scan were selected for fragmentation by higher-energy collisional dissociation.

### 4.9. Cellular Fractionation

Cytoplasmic and mitochondrial enrichments were collected using the mitochondrial isolation kit for tissue (Abcam, Cambridge, UK). RV specimens were homogenized with a Dounce homogenizer in isolation buffer supplemented with mammalian protease inhibitor (Sigma-Aldrich) and 100 µM PUGNAc (Sigma-Aldrich). After completing the centrifugation steps, the supernatant (cytoplasm) was saved for analysis. The final mitochondrial pellet was resuspended in SDS buffer [31] supplemented with protease inhibitor (Sigma-Aldrich). Protein concentration was determined using a BCA kit and equivalent amounts of protein were subjected to Western blot analysis as described above.

### 4.10. Cell Culture

*Rattus norvegicus*, cardiac myoblast cell line H9c2 was a gift from Dr. Jin O-Uchi. Cells were maintained in Dulbecco’s modified Eagle’s medium with 4500 g/L glucose (DMEM) (Thermo Scientific), supplemented with 10% fetal bovine serum (Equitech Bio, Kerrville, TX, USA), 100 Units/mL penicillin, and 100 µg/mL streptomycin (Invitrogen, Carlsbad, CA, USA). Cells were passaged with Accutase (Biolegend, San Diego, CA, USA). To examine the effects of colchicine, 200,000 cells were plated per well of a 48-well dish with or without 100 nM colchicine. The following day, protein was collected in Laemmli sample buffer supplemented with HALT protease and phosphatase inhibitor cocktail (ThermoFisher).

### 4.11. Mitochondrial Respiration Measurements

H9c2 cells were seeded at a density of 20,000 cells per well in Agilent (Santa Clara, CA, USA) Seahorse XFp cell culture plates. One hour after plating, the media was changed to either control DMEM or DMEM supplemented with 100 nM colchicine (Sigma). The following day and 1 h before the assay, cell media was changed to Seahorse DMEM assay media and supplemented with 5 mM glucose, 4 mM glutamine, 1 mM pyruvate (Agilent, Santa Clara, CA, USA). Oxygen consumption rate (OCR) was measured with an Agilent Seahorse XFp Extracellular Flux analyzer using the Agilent XFp Cell Mito Stress Test Kit with oligomycin A (final concentration after injection 1.5 µM), carbonyl cyanide 4-(trifluoromethoxy)phenylhydrazone (FCCP, 2 µM), and rotenone/actimycin A (0.5 mM). After the assay, cells were detached with Accutase and resuspended in equal amounts of PBS solution and 0.4% trypan blue. Cell well counts were estimated by multiplying initial cell number times final cell viability as measured by the EVE automated cell counter (NanoEnTek, Seoul, South Korea). Raw measurements were normalized to viable cell number and also baselined to non-mitochondrial respiration for multiplate comparison using Wave software (Agilent). ATP-linked respiration, proton leak, and spare respiratory capacity was obtained as per manufacturer’s definitions.

### 4.12. PAH Patient Cohort

PAH patients from the University of Minnesota Pulmonary Hypertension Program [43] were analyzed. PAH was defined as mean pulmonary arterial pressure (mPAP) >20 mmHg, pulmonary capillary wedge pressure <15 mmHg, and pulmonary vascular resistance (PVR) >3.0 Wood units, with other causes such as left sided heart disease, chronic lung disease, or chronic thromboembolic disease being ruled out using echocardiography, pulmonary function tests, computed tomography imaging, ventilation perfusion imaging, or invasive pulmonary angiogram [44]. Only PAH patients with a right heart catheterization performed after 2015 could be included as older studies did not have the raw data from the RV pressure tracings. We excluded scleroderma-associated PAH due to their disproportionate RV dysfunction [45]. We assessed 45 PAH patients, 73% were female, and average age was 53.7 years old. The cohort comprised 31 nondiabetic and 14 diabetic PAH patients. Hemoglobin A1C levels were collected as clinically indicated. All patients signed a consent form to participate in the study.

### 4.13. RV Contractility Analysis

RV contractility was estimated by calculating *dp/dt*_max_/instantaneous pressure from RV pressure tracings from right heart catheterization procedures using a custom-made program (LabView) as previously described [46].

### 4.14. Quantitative Immunofluorescence of Human RV Samples

Endomyocardial biopsy samples were collected from PAH patients as previously described [47], and all patients signed a consent form to be in the study. Deparaffinized and rehydrated sections were incubated with fluorescein labeled succinylated wheat germ agglutinin (Vector Laboratories, Burlingame, CA, USA) (1:50) and Alexa 633-WGA overnight at 4 °C, washed in PBS twice, treated with autofluorescence quenching kit (Vector Laboratories), and mounted in Antifade media containing DAPI (Vector Laboratories). Confocal images were collected using a Bio-Rad (Hercules, CA, USA) MRC 1000 scan head mounted on an upright Nikon Optishot (Tokyo, Japan) microscope at the University of Minnesota Imaging Center. FIJI (National Institutes of Health, Bethesda, MD, USA) was used to quantify total intracellular succinylated WGA signal normalized to area.

### 4.15. Relationship Between HBP Intermediates, O-GlcNAcylation, and RV Function

We performed correlational analysis between the RV levels of UDP-GlcNAc and RV levels of O-GlcNAcylation as determined by Western blot and echocardiography defined TAPSE and cardiac output for individual animals.

### 4.16. Statistical Analysis

To compare means of two groups, *t*-test was used if the variance was similar but if there was difference in variance as determined by F-test, Mann–Whitney U-test was used. When comparing the means of three groups, one-way analysis of variance (ANOVA) with Tukey post-hoc analysis was implemented. To analyze global changes in proteomic samples, principal component analysis (PCA) and heat maps were generated using MetaboAnalyst [48]. In metabolomics analysis, the PCA and heat maps were generated independently by Metabolon. To understand the cellular function of O-GlcNAcylated proteins, we performed functional enrichment analysis (Toppgene website [49]). The most differentially O-GlcNAcylated proteins was determined by *p*-value calculated by Benjamini–Hochberg procedure on Scaffold software. Statistically significant was defined as a *p*-value <0.0119. All statistical analysis and graphing were performed on Prism except for the PCA and heat map analysis as described above. Values are presented as mean ± standard deviation. Graphs are depicted as Violin plots to display the distribution of the data.

## 5. Limitation

Our study has important limitations that need to be acknowledged. First, it is possible that AMP kinase activation promotes improved mitochondrial function independent of O-GlcNAcylation as AMP-kinase activation promotes mitochondrial biogenesis [50]. Second, we excluded scleroderma PAH (SSc-PAH) patients from our analysis because they have worse RV function at baseline than idiopathic PAH patients [45]. There is evidence that SSc-PAH patients exhibit RV capillary rarefaction [17] and hypoxia can induce expression of GFAT [51], which could then further promote O-GlcNAcylation. Unfortunately, we did not have enough tissue from SSc-PAH to perform this analysis. The beneficial effects of colchicine on RV function in MCT rats may be multifactorial rather than solely alteration of O-GlcNAcylation as colchicine has anti-inflammatory and microtubule depolymerizing effects [52]. However, we did not see any improvements in RV function in PAB rats treated with colchicine (Figure 2). The metabolomic changes observed in the MCT rats treated with colchicine may also be due to reduction in RV afterload as we previously showed colchicine can blunt pulmonary vascular remodeling [31]. However, we showed colchicine directly augmented mitochondrial function in H9c2 cells, suggesting that the metabolic shifts in the MCT rats treated with colchicine could be due to direct augmentation of mitochondrial capacity by colchicine. The difference in RV contractility in PAH patients with diabetes is likely multifactorial and not solely due to O-GlcNAcylation. Finally, we do not routinely collect HgbA1C in our PAH patients, so we were unable to analyze the relationship between HgbA1C and RV contractility in all of our PAH patients. The proteomics experiments may have identified proteins that were not directly O-GlcNAcylated as some proteins may have interacted with proteins pulled down with the RL2 antibody. Likewise, we only used the RL2 antibody and not the CTD110.6 antibody to quantify O-GlcNAcylation. Finally, other cell types in RV extracts could have contributed to the changes in O-GlcNAcylation and metabolomics.

## Figures and Tables

**Table 1:** Summary of top 10 mitochondrial proteins identified in proteomics experiment.

Abbreviation	Protein	Control Levels (*n* = 3)	MCT Levels (*n* = 3)	MCT-Colch Levels (*n* = 4)	Previously Identified as O-GlcNAcylated	Citation
HADHA	Trifunctional enzyme subunit alpha	1.0 ± 0.1	2.0 ± 1.2	1.2 ± 0.1	Yes	[14,24]
SLC25A3	Phosphate carrier protein	1.0 ± 0.1	1.6 ± 0.3	1.4 ± 0.2	Yes	[14,24]
CHCHD3	MICOS complex subunit	1.0 ± 0.2	1.6 ± 0.2	1.3 ± 0.2	Yes	[14,24]
ATP5F1A	ATP synthase subunit alpha	1.0 ± 0.1	1.3 ± 0.2	1.2 ± 0.2	Yes	[14,24]
IMMT	MICOS complex subunit MIC60	1.0 ± 0.1	1.4 ± 0.2	1.1 ± 0.1	Yes	[14,24]
HADHB	Trifunctional enzyme subunit beta	1.0 ± 0.1	1.9 ± 1.1	1.1 ± 0.1	Yes	[14,24]
NDUFS1	NADH-ubiquinone oxidoreductase 75 kDa subunit	1.0 ± 0.1	1.3 ± 0.1	1.3 ± 0.2	Yes	[14,24]
UQCRC2	Cytochrome b-c1 complex subunit 2	1.0 ± 0.2	1.5 ± 0.5	1.5 ± 0.3	Yes	[14,24]
MRPL34	Mitochondrial ribosomal protein L34	1.0 ± 0.3	3.2 ± 0.5	2.5 ± 0.4	No	NA
SUCLG2	Succinate-CoA ligase subunit beta	1.0 ± 0.2	1.5 ± 0.5	1.5 ± 0.4	No	NA

**Table 2:** Clinical characterization of PAH patients.

Characteristics	Total Cohort (*n* = 45)	Nondiabetic (*n* = 31)	Diabetic (*n* = 14)	*p*-Value
Female, *n* (%)	33 (73)	23 (75)	10 (71)	1.0
Age	53.7 ± 16.0	50.0 ± 15.5	61.9 ± 14.3	0.019
BMI, kg/m^2^	31.6 ± 8.1	30.3 ± 8.0	34.7 ± 7.9	0.089
**WHO Group 1 Etiology *n* (%)**				
Associated	23 (51)	15 (48)	8 (57)	0.75
Idiopathic	17 (38)	13 (42)	4 (29)	0.51
Drug/toxin	4 (9)	3 (10)	1 (7)	1.0
Heritable	1 (2)	0 (0)	1 (7)	0.31
**Medications, *n* (%)**				
Oxygen	12 (27)	9 (29)	3 (21)	0.73
Diuretics	30 (67)	19 (61)	11 (79)	0.32
Digoxin	3 (7)	1 (3)	2 (14)	0.22
Warfarin	5 (11)	3 (10)	2 (14)	0.64
Calcium channel blockers	4 (9)	2 (6)	2 (14)	0.58
Phosphodiesterase-5-inhibitors	17 (38)	12 (39)	5 (36)	1.0
Endothelin receptor antagonists	8 (18)	7 (23)	1 (7)	0.40
Prostacyclins	7 (16)	6 (19)	1 (7)	0.41
**Six minute walk test**				
Distance, meters	333 ± 157	368 ± 146	200 ± 133	0.03
Rest oxygen saturation, %	96 ± 2	96 ± 2	96 ± 2	0.53
Peak exercise oxygen saturation, %	91 ± 4	90 ± 5	91 ± 4	0.70
Borg dyspnea score	4 ± 1	4 ± 2	5 ± 1	0.33
**Hemodynamics**				
Heart rate, beats/min	76 ± 15	77 ± 17	74 ± 9	0.47
Mean right atrial, mm Hg	9 ± 5	8 ± 6	10 ± 4	0.37
Mean PAP, mm Hg	48 ± 14	48 ± 16	47 ± 12	0.80
PCWP, mm Hg	10 ± 3	10 ± 3	10 ± 4	0.60
Cardiac output, L/min	5.1 ± 1.9	5.1 ± 2.0	5.3 ± 2.0	0.72
Cardiac Index, L/min/m^2^	2.6 ± 1.0	2.6 ± 1.0	2.6 ± 2.0	0.99
PVR, WU	8.9 ± 5.5	9.2 ± 5.5	8.6 ± 5.8	0.75
PAC, mL/mm Hg	1.8 ± 1.1	1.8 ± 1.2	1.9 ± 1.1	0.74
**Measures of RV Function**				
NT pro-BNP, pg/mL	1529 ± 2454	1125 ± 1279	2737 ± 3969	0.16
TAPSE, cm	1.9 ± 0.5	1.9 ± 0.4	1.9 ± 0.6	0.96
RVFAC, %	33 ± 9	33 ± 10	31 ± 7	0.49
S’, cm/s	10.6 ± 2.3	10.7 ± 2.4	9.9 ± 1.8	0.44
RVEDP, mm Hg	11 ± 6	10 ± 6	12 ± 5	0.58

## Supplementary Materials

The following are available online at https://www.mdpi.com/1422-0067/21/19/7278/s1.
