# Supplementary material for: Excess Protein O-GlcNAcylation Links Metabolic Derangements to Right Ventricular Dysfunction in Pulmonary Arterial Hypertension
Source: Int J Mol Sci. 2020 Oct 1;21(19):7278. doi: 10.3390/ijms21197278 (PMC7582480; doi:10.3390/ijms21197278)
Supplement: Supplementary file 1 [file ijms-21-07278-s001.zip › ijms-953352 Supplemental.docx]

**Supplemental Figure 1:** Comparison of the RV phenotype of MCT and PAB rats. (A) Fulton index to quantify right ventricular hypertrophy (Control: 0.27±0.05, MCT: 0.70±0.6, Sham: 0.30±0.03, PAB: 0.52±0.09). (B) TAPSE (Control: 2.9±0.5, MCT: 1.7±0.3, Sham: 3.1±0.2, PAB: 2.7±0.2). (*) indicates different from sham, (#) indicates different from control, and (&) indicates different from PAB as determined by one-way *ANOVA* with Tukey post-hoc analysis. *N*=8-10 animals per group. Analysis performed four weeks after MCT injection and eight weeks after sham or PAB procedure.

**
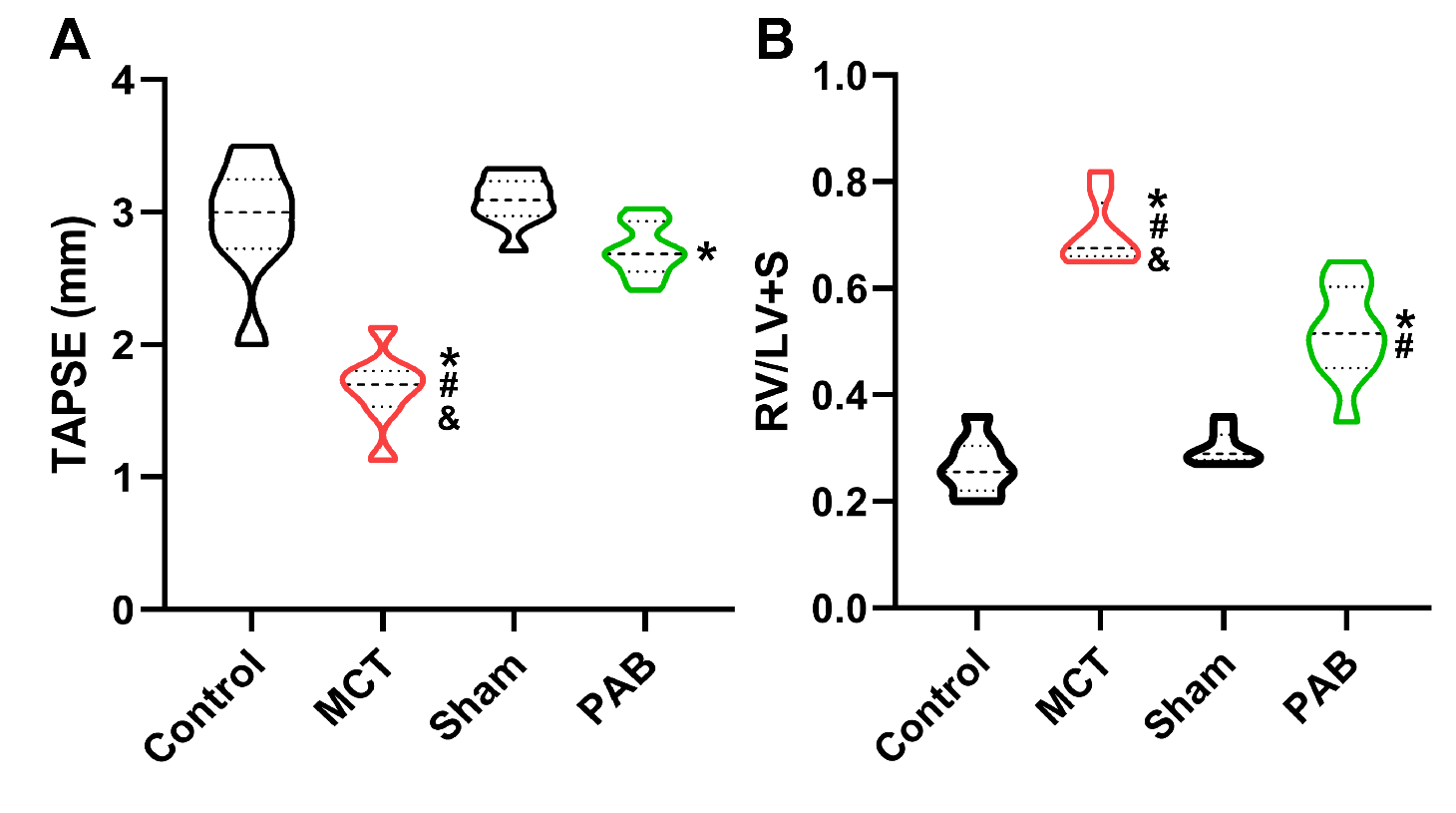
**

**Supplemental Figure 2:** Examination of O-GlcNAcylation in the left ventricle of MCT and PAB rats. (A) Representative Western blots and quantification of protein abundance (B) in MCT LV extracts. (C) Representative Western blots and quantification of protein abundance (D) in PAB LV extracts. There was no difference in O-GlcNAcylation and GFAT1 in MCT and PAB LVs, but both models exhibited downregulation of OGT and OGA. *n*=4-5 animals per group. Blots were performed on tissue collected four weeks after MCT injection.

**
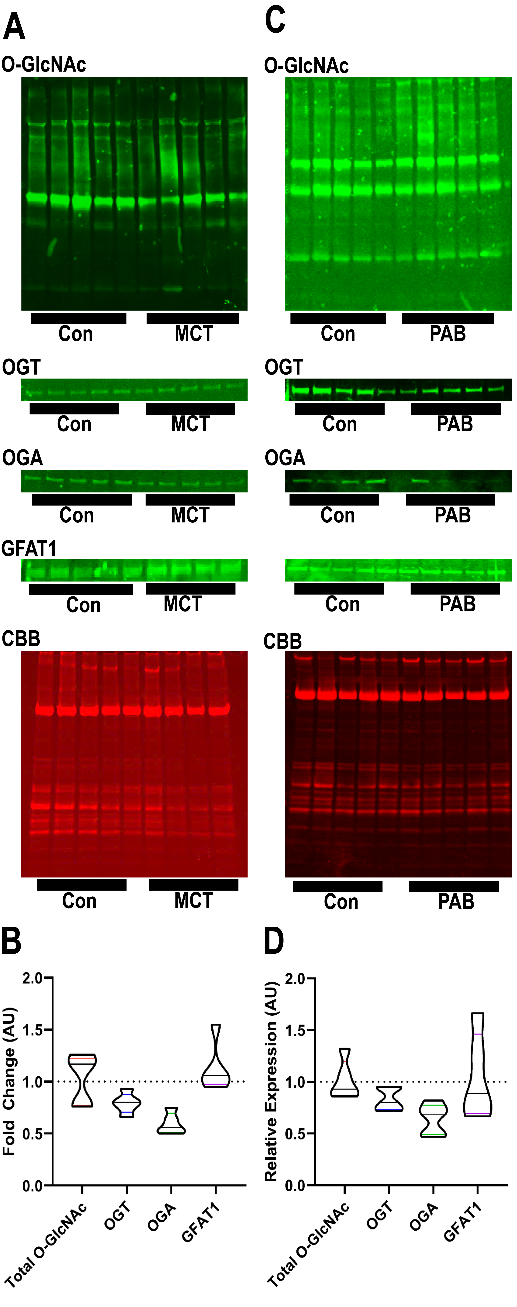
**

**Supplemental Figure 3:** Summary of MCT metabolomics study. (A) Table depicting the overall differences in metabolites in control, MCT, and MCT-Colch RVs. (B) Biochemical importance plot revealing the top 30 ranking biochemicals that differentiate experimental groups. (C) Random Forest Confusion Matrix shows analysis of metabolomics data results in an 86% predictive accuracy when classifying rats. Tissue was harvest four weeks after MCT injection.


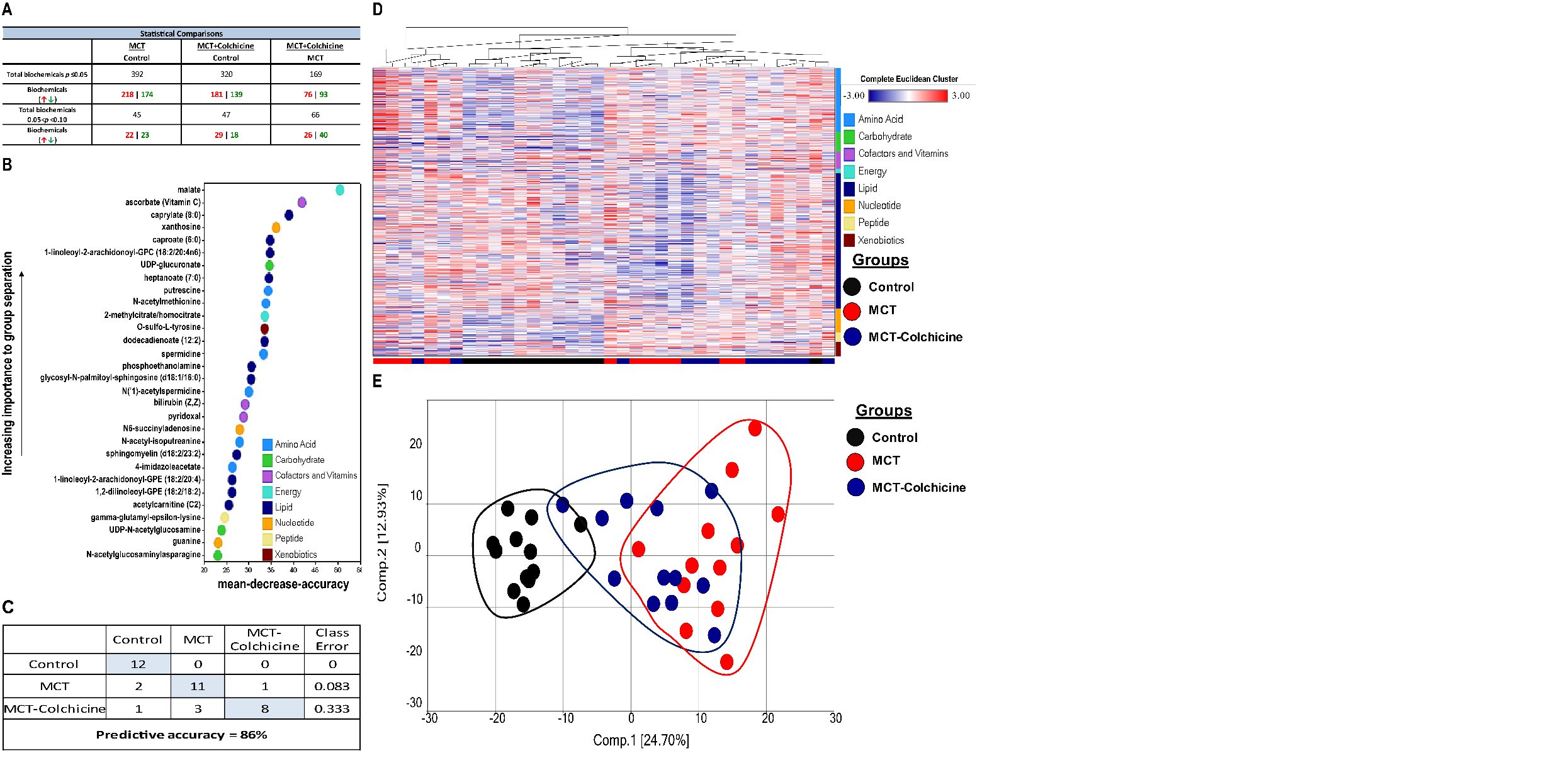


**Supplemental Figure 4** Examination of O-GlcNAcylation in the cytoplasmic and mitochondrial fractions of control, MCT, and MCT-Colch RVs. (A) Coomassie brilliant blue stained SDS-PAGE from cytoplasmic and mitochondrial extracts (upper) and representative Western blot of O-GlcNAcylation in extracts (lower). The mitochondrial protein mitofusin-2 is enriched in the mitochondrial fraction while α-tubulin is only found in the cytoplasmic fraction. (B) Quantification of total O-GlcNAcylation in the cytoplasmic and mitochondrial fractions. There were minimal changes in cytoplasmic O-GlcNAcylation (MCT: 1.1±0.2 fold increase, MCT:Colch: 1.1±0.5 fold increase) and increases in mitochondrial O-GlcNAcylation in MCT RVs (MCT: 2.0±0.5 fold increase, MCT-Colch:1.3±0.4 fold increase). Colchicine treatment reduced mitochondrial O-GlcNAcylation. *N*=3 animals per group in fractionation study. Blots were performed on tissue collected four weeks after MCT injection.


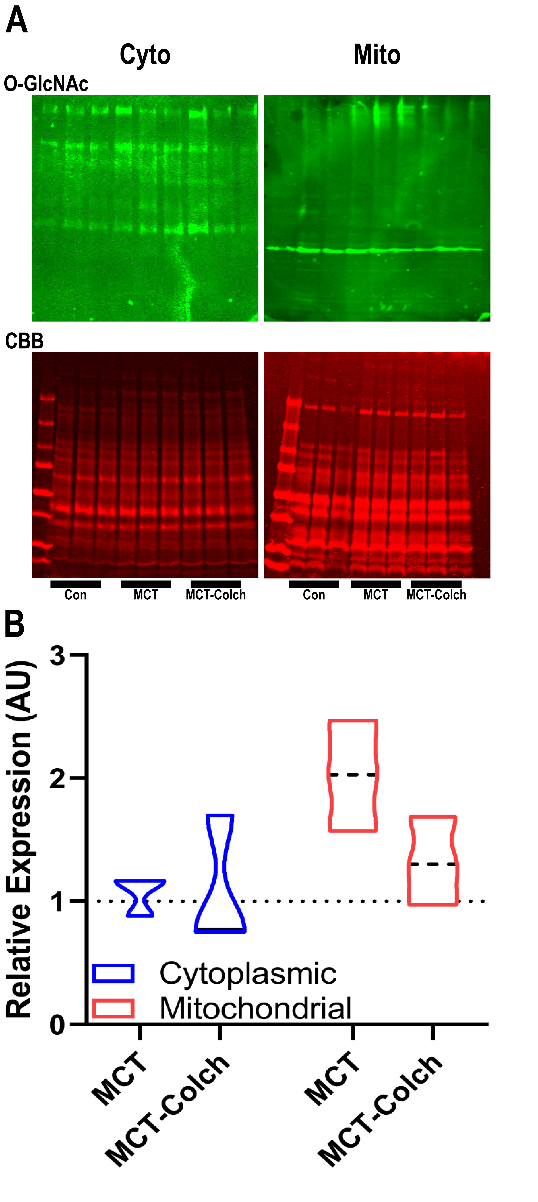


**Supplemental Figure 5:** Colchicine directly augments mitochondrial function in H9c2 cardiomyocytes. (A) Representative oxygen consumption rate (OCR) analysis in control and colchicine-treated cells (100 nM). (B) Representative ECAR analysis from control and colchicine treated cells. Colchicine treatment significantly increased maximal OCR (Colch: 532.6±103.6 vs Control: 445.6±126.7, *p*=0.03) (C) and spare capacity (Colch: 352.3±85.2 vs Control: 251.3±96.5, *p*=0.001) (D) while significantly decreased proton leak (Colch: 27.0±18.2 vs Control: 50.5±29.5, *p*=0.0005) (E), all indicative of augmented mitochondrial function. N=19-20 cell wells per group. (F) Representative Western blots and quantification (G) of O-GlcNAcylation (0.8±0.3 expression relative to control), AMPK (1.3±0.4 fold increase), pAMPK (1.8±0.3 fold increase), pAMPK/AMPK (1.5±0.5 fold increase), OGT (0.85±0.24 relative expression), OGA (0.79±0.28 relative expression), and GFAT1 (1.1±0.2 fold increase) in H9c2 cells incubated without or with 100 nM colchicine.(*) indicates significantly different from control as determined by *t*-test.


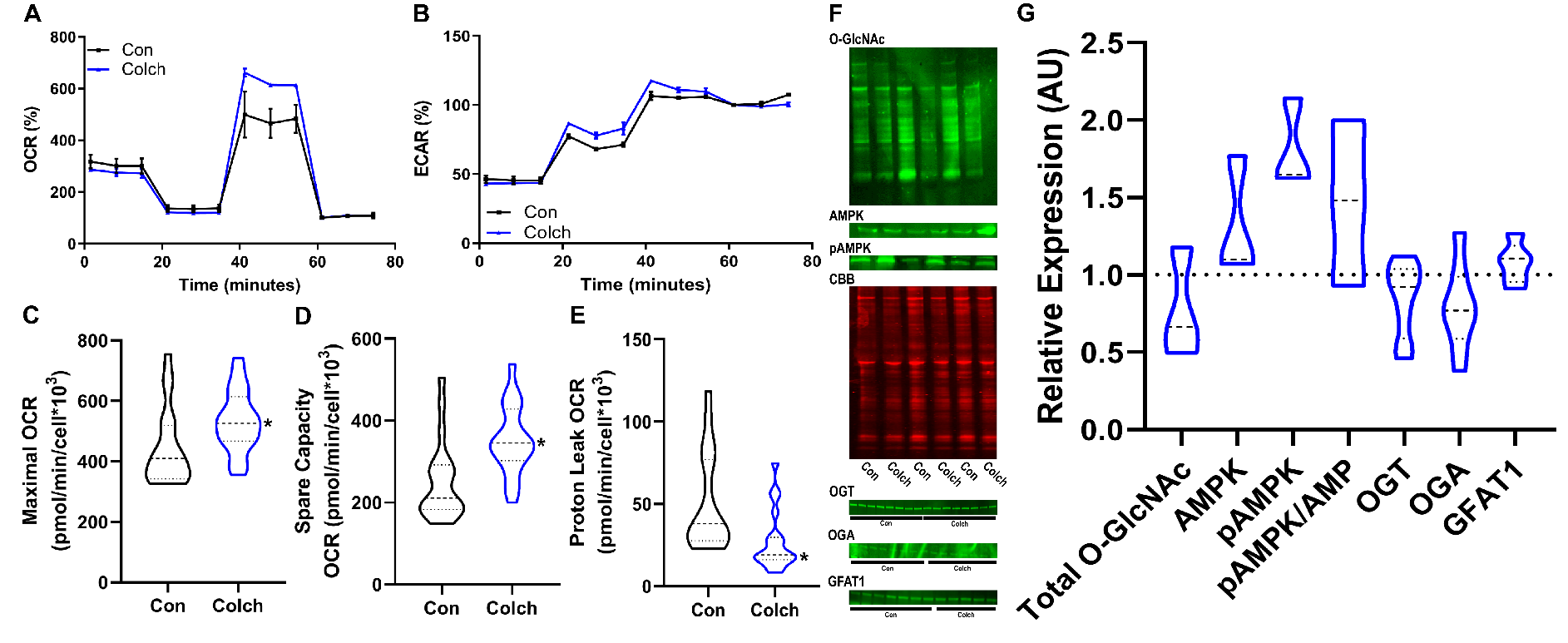


**Supplemental Figure 6**: Colchicine reduces RV microtubule content in PAB rats. (A) Representative Western blots and quantification (B) of α-tubulin (1.6±0.2 fold increase), β-tubulin (1.5±0.4 fold increase), and detyrosinated α−tubulin (1.9±0.5 fold increase) in the RV of PAB rats and α-tubulin (1.0±0.4 fold increase), β-tubulin (1.1±0.3 fold increase), and detyrosinated α-tubulin (1.0±0.5 fold increase) in the RV of PAB-Colch rats. *N*=5 animals per group. Blots were performed on tissue collected eight weeks after sham or PAB procedure.


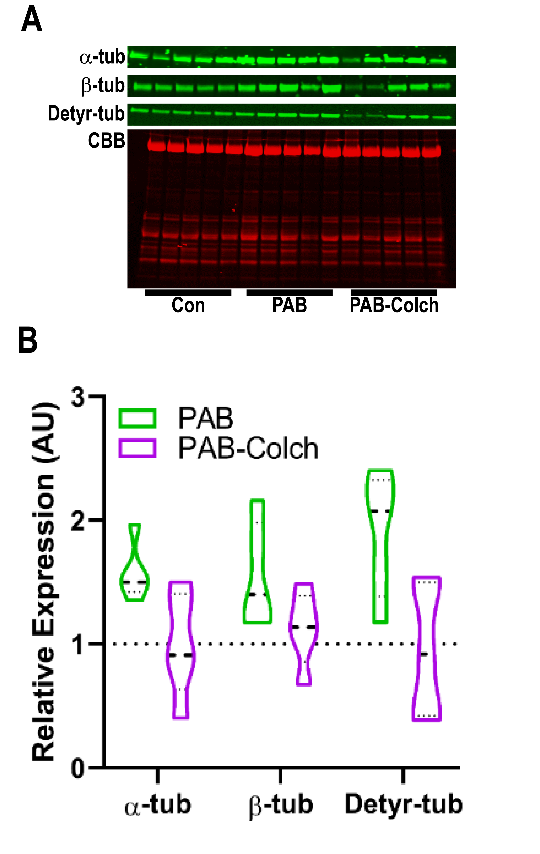


**Supplemental Table 3:** Antibodies Used in Study

| **Antigen** | **Company** | **Species** | **Catalogue Number** | **Dilution** |
| --- | --- | --- | --- | --- |
| O-GlcNAc (Western Blot) | ThermoScientific | Mouse | MA1-072 | 1:100 |
| O-GlcNAc (Immunofluorescence) | ThermoScientific | Mouse | MA1-072 | 1:50 |
| OGT | Abcam | Rabbit | Ab177941 | 1:250 |
| OGA | Abcam | Rabbit | Ab124807 | 1:100 |
| GFAT | Abcam | Rabbit | Ab176775 | 1:250 |
| Phosphorylated GFAT | Previously published[1] | Mouse | NA | 1:500 |
| AMP Kinase | Cell Signaling Technology | Mouse | 2795 | 1:250 |
| Phosphorylated AMP Kinase | Cell Signaling Technology | Rabbit | 2531 | 1:250 |
| α-tubulin | Millipore | Mouse | 05-829 | 1:500 |
| β-tubulin | Sigma-Aldrich | Mouse | T402 | 1:250 |
| Detyrosinated α-tubulin | Abcam | Rabbit | Ab48389 | 1:500 |
| Mitofusin-2 | Abcam | Mouse | Ab56889 | 1:250 |
| Anti-mouse secondary | Li-COR | Goat | 926-68070 | 1:10,000 |
| Anti-rabbit secondary | Li-COR | Goat | 926-32210 | 1:10,000 |
| Anti-mouse secondary | ThermoFIsher | Goat | A32723 | 1:500 |

1. Moloughney, J. G., N. M. Vega-Cotto, S. Liu, C. Patel, P. K. Kim, C. C. Wu, D. Albaciete, C. Magaway, A. Chang, S. Rajput*, et al.* "Mtorc2 modulates the amplitude and duration of gfat1 ser-243 phosphorylation to maintain flux through the hexosamine pathway during starvation." *J Biol Chem* 293 (2018): 16464-78. 10.1074/jbc.RA118.003991. <https://www.ncbi.nlm.nih.gov/pubmed/30201609>.
